# *Chlamydia trachomatis* Infection Leads to Defined Alterations to the Lipid Droplet Proteome in Epithelial Cells

**DOI:** 10.1371/journal.pone.0124630

**Published:** 2015-04-24

**Authors:** Hector Alex Saka, J. Will Thompson, Yi-Shan Chen, Laura G. Dubois, Joel T. Haas, Arthur Moseley, Raphael H. Valdivia

**Affiliations:** 1 Department of Molecular Genetics and Microbiology and Center for Microbial Pathogenesis, Duke University Medical Center, Durham, North Carolina, United States of America; 2 Proteomics and Metabolomics Core Facility, Duke University Medical Center, Durham, North Carolina, United States of America; 3 Department of Biochemistry and Biophysics, UCSF, San Francisco, California, United States of America; University of Würzburg, GERMANY

## Abstract

The obligate intracellular bacterium *Chlamydia trachomatis *is a major human pathogen and a main cause of genital and ocular diseases. During its intracellular cycle, *C*. *trachomatis *replicates inside a membrane-bound vacuole termed an “inclusion”. Acquisition of lipids (and other nutrients) from the host cell is a critical step in chlamydial replication. Lipid droplets (LD) are ubiquitous, ER-derived neutral lipid-rich storage organelles surrounded by a phospholipids monolayer and associated proteins. Previous studies have shown that LDs accumulate at the periphery of, and eventually translocate into, the chlamydial inclusion. These observations point out to *Chlamydia*-mediated manipulation of LDs in infected cells, which may impact the function and thereby the protein composition of these organelles. By means of a label-free quantitative mass spectrometry approach we found that the LD proteome is modified in the context of *C*. *trachomatis* infection. We determined that LDs isolated from *C*. *trachomatis-*infected cells were enriched in proteins related to lipid metabolism, biosynthesis and LD-specific functions. Interestingly, consistent with the observation that LDs intimately associate with the inclusion, a subset of inclusion membrane proteins co-purified with LD protein extracts. Finally, genetic ablation of LDs negatively affected generation of *C*. *trachomatis* infectious progeny, consistent with a role for LD biogenesis in optimal chlamydial growth.

## Introduction

The obligate intracellular bacterium *Chlamydia trachomatis* is a major human pathogen and the causative agent of genital and ocular diseases. *C*. *trachomatis* is the leading cause of sexually transmitted infections (STIs) with more than 105 million new cases per year according to global estimates [1]. A high proportion of chlamydial STIs are asymptomatic and thereby left untreated, favoring both the transmission and the occurrence of serious complications like pelvic inflammatory disease, infertility and ectopic pregnancies [2, 3]. In the United States, direct medical costs of genital infections caused by *C*. *trachomatis* have reached costs as high as half a billion dollars annually [4]. This bacterium is also the etiologic agent of trachoma, an ocular disease endemic in 56 countries and the main cause of infectious blindness. Globally, more than 40 million people suffer from active trachoma, 8.2 million are affected by trichiasis, 1.8 million have low vision and 1.3 million are blind, producing an economic cost of nearly 8 billion US dollars ([5] and references therein).

*C*. *trachomatis*, as well as other species belonging to *Chlamydiaceae*, replicate exclusively within eukaryotic cells and display a unique life cycle alternating between two distinct developmental forms. The elementary body (EB) is the infectious form and is characteristically smaller and environmentally stable. Soon after attachment to epithelial cells, EBs are internalized and confined to a vacuole termed an “inclusion” in a process that requires the secretion of bacterial effectors and reprogramming of the host cell’s endocytic machinery [6]. At early stages post-infection EBs transition to the replicative form called the reticulate body (RB), which actively divides by binary fission, is larger than the EB form and is non-infectious. As the inclusion expands, RBs begin to transition back to the EB form in an asynchronous process ([7] and references therein). At the end of the cycle, the inclusion occupies most of the host cell’s cytoplasm and the EBs are finally released to the extracellular space through cell lysis or extrusion of intact inclusions [8], allowing the initiation of new cycles of infection. During its intracellular cycle, *Chlamydia* manipulates the host cell’s membrane trafficking pathways to avoid lysosomal destruction [9, 10]. Because *Chlamydia* lacks the ability to synthesize many essential metabolites [11], acquisition of nutrients from their hosts, including lipids, is a critical step in chlamydial biology and survival. One proposed mechanism includes the re-routing of Golgi-derived exocytic vesicles and multivesicular bodies to deliver host-derived glycerophospholipids, sphingolipids and cholesterol to the inclusion [12–17]. In addition, a subset of Rab GTPases linked to ER-Golgi trafficking and endosomal functions is recruited to the inclusion and may play a role in lipid acquisition by *Chlamydia*. For instance, Rab6, Rab11 and Rab14 have been implicated in sphingomyelin acquisition, likely by facilitating the fusion of selected lipid-containing host vesicles with the inclusion [18–20]. In addition, *Chlamydia* co-opts non-vesicle-mediated pathways for lipid acquisition. Incorporation of host glycerophospholipids, like phosphatidylinositol and phosphocholine, may occur through non-vesicle mediated transport pathways in a process involving host Ca^2+^-dependent cytosolic phospholipase A2 and activation of ERK [12, 21, 22]. More recent studies provide evidence that *C*. *trachomatis* inclusion membrane protein IncD is a binding partner for the cytosolic lipid transport protein CERT, which in turn recruits the endoplasmic reticulum (ER)-resident protein VAPB [23, 24]. CERT normally associates with VAPA and VAPB in ER-Golgi membrane contact sites and mediates the transfer of ceramide from ER to Golgi [25–27].

Previous studies indicated that lipid droplets (LD) are targeted by *Chlamydia* during its intracellular stage of replication [28–30]. Lipid droplets are ubiquitous, ER-derived neutral lipid-rich organelles, composed by a core of acylglycerols (mainly triacylglycerols) and sterol esters surrounded by a phospholipids monolayer [31]. Although LDs were traditionally considered to be relatively inert, recent work by multiple groups now implicates LDs in a wide range of functions (reviewed in [32]), including those related to host-pathogen interactions (reviewed in [33]). We previously identified at least two chlamydial proteins, Lda1 and Lda3, that localized to LDs when ectopically expressed in HeLa cells [28]. In addition, LDs accumulated at the periphery of the inclusion and the neutral lipid content increased in epithelial cells infected with *C*. *trachomatis*. Moreover, pharmacological inhibition of neutral lipid synthesis impaired *C*. *trachomatis* growth. Unexpectedly, LDs can be translocated into the lumen of the chlamydial inclusion, although the significance of these phenomena is unclear [30]. These observations lead us to predict that *Chlamydia* alters the function of LDs in infected cells, a process that should be reflected by defined changes in the protein composition of these organelles. This study describes a quantitative proteomic characterization of LDs isolated from *C*. *trachomatis*-infected and uninfected cells. We used bottom-up LC-MS/MS with data-independent and data-dependent acquisition to apply area-under-the-curve label-free quantification of proteins. We then estimated protein abundance (in fmol μg^-1^) in the LD based on average intensity of the top 3 peptides per protein relative to a known internal standard [34, 35]. Consistent with the observed increase in neutral lipid content in infected cells, we determined that LDs isolated from *C*. *trachomatis*-infected cells at 40 hours post-infection (hpi) were enriched in proteins involved in lipid metabolic processes, lipid biosynthetic processes and LD-specific functions. In addition, we found a subset of inclusion membrane proteins co-purifying with LDs, consistent with the notion that *C*. *trachomatis* inclusion intimately associates with LDs.

## Materials and Methods

### *C*. *trachomatis* strain, cell lines, culture conditions and infections

*C*. *trachomatis* LGV-L2 434/Bu used in this study was propagated in HeLa CCL2 (ATCC, Rockville, Maryland, USA). Mouse embryonic fibroblasts (MEF) derived from either wild type or diacylglycerol-acyltransferases 1 and 2 (DGAT1 and DGAT2) double knock-out animals were isolated and immortalized as described previously [36]. Briefly, E14.5 embryos were collected by cesarean dissection and prepared as MEFs after removing the head and hematopoietic sac. For complementation, MEFs were transduced with MSCV-FLAG-hDGAT1 as described previously [37]. HeLa and MEF cells were both grown in Dulbecco’s minimal essential medium (DMEM high glucose, Gibco/ Invitrogen Life Technologies, Carlsbad, California, USA) supplemented with 10% fetal bovine serum (Mediatech, Manassas, Virginia, USA) at 37 °C, 5% CO_2_ in a humidified atmosphere. For induction of LD formation, oleic acid (OA, Sigma) was pre-complexed with cell culture-tested, fatty acid-free BSA (Sigma) in PBS and briefly emulsified by sonication. OA was added to growth media at final concentrations ranging 25–400 μM for 8–14 h before harvesting or fixation, as indicated. Infections for large-scale preparation of LDs were carried out by adding a suspension of density gradient-purified *C*. *trachomatis* LGV-L2 434/Bu EBs at a multiplicity of infection (MOI) of 10 at time zero and incubated for either 20h or 40h, as indicated. Infections for all other experiments were done by adding a suspension of purified EBs at an MOI of 0.5–1 in culture media followed by centrifugation at 3,000x*g* for 25 min at 10 °C. Then infected cells were transferred to the tissue-culture incubator for 15 min, washed once with PBS, replenished with fresh media and returned to the tissue culture incubator for the indicated hpi.

### LD purification from infected and uninfected cells

For LD purification, HeLa cells were grown in 8x150 mm dishes per experimental condition (uninfected, infected 20 hpi and infected 40 hpi). Lipid loading was induced by addition of OA 100 μM for 14 h before harvesting. Because the 20 hpi and 40 hpi experiments were carried out about 3 weeks apart, uninfected controls for each experiment were performed independently. LDs were purified as described elsewhere [30] with minor modifications. Briefly, lipid loaded monolayers were washed three times with ice-cold PBS and collected in 5 ml TNE buffer [20 mM Tris-HCl (pH 7.5), 0.15 M NaCl, and 1 mM EDTA] containing protease inhibitors (Roche Diagnostics). Cell lysis was carried out on ice with 30 strokes/150 mm dish in a Dounce homogenizer and a small fraction of total lysates (80 μl) was collected from each sample and stored at -80°C for further processing. Cell lysates were then adjusted to 0.45 M sucrose, overlaid with 2 ml each of 0.25 M Sucrose/TNE, 0.15 M Sucrose/TNE, and TNE buffer alone and centrifuged at 30,000 rpm for 90 min at 4°C in an SW41 rotor (Beckman Coulter), which was allowed to coast to a stop. The floating LD-enriched fat layer was gently collected and diluted in TNE buffer for washing and refloated at 47,000 rpm for 45 min in a TLA55 rotor at 4°C (Beckman Coulter). Refloated LDs were collected and lipids were extracted with 4 volumes of diethyl ether. Delipidated proteins were acetone precipitated for 1 h on ice and solubilized in 0.1% RapiGest SF Surfactant [Waters Corp., Milford, Maryland, USA] in 100 mM NH_4_HCO_3_ for preparation of extracts for proteomics analysis or immunoblots.

### Immunoblots

Antibodies used were: ACSL3 (rabbit polyclonal, Abcam), ACSL4 (rabbit polyclonal, ProteinTech Group), Aup-1 (rabbit polyclonal, ProteinTech Group), Calreticulin (rabbit polyclonal, Stressgen), FAF2 (mouse polyclonal, Abnova), GAPDH (rabbit polyclonal, Abcam), Integrinα2 (mouse monoclonal, BD Biosciences), NSDHL (rabbit polyclonal, Abcam), PLIN2 (mouse monoclonal, ProGen Biotechnik), PLIN3 (rabbit polyclonal, ProteinTech Group), PLPL2 (rabbit polyclonal, Abcam), Rab1 (rabbit polyclonal, Santa Cruz Biotechnology), Rab11 (mouse monoclonal, BD Biosciences), RpoB (rabbit polyclonal, kindly provided by M. Tan, UC Irvine), TRAPα (mouse monoclonal, Abcam). For immunoblots, protein extracts were normalized for total protein content by Bradford assay [38] and equal amounts per lane were separated by SDS-PAGE in 4–15% pre-casted gradient gels (Bio-Rad, Hercules, California, USA) and transferred to 0.45 μm nitrocellulose membranes by means of a Trans-Blot SD Semi-Dry Electrophoretic Transfer Cell (Bio-Rad, Hercules, California, USA). Nitrocellulose membranes were blocked in 5% non-fat powder milk in TBST (50 mM Tris-Base, 150 mM NaCl, 0.1% Tween 20 pH 7.4), incubated with primary antibodies appropriately diluted in 5% non-fat powder milk in TBST, washed in TBST and then incubated with goat anti-rabbit or anti-mouse secondary antibodies conjugated to horseradish peroxidase (Sigma-Aldrich, St. Louis, Missouri, USA). Chemoluminescence reaction (Pierce, Rockford, Illinois, USA) and ECL-hyperfilm (GE Healthcare, Princeton, New Jersey, USA) were used to detect immunoreactive material.

### Generation of constructs for ectopic expression of LD-associated proteins and transfections

Full length Cap1 (CTL0791/CT529), CTL0882 (CT618) and IncG (CTL0373/CT118) were PCR amplified from genomic DNA of *C*. *trachomatis* LGV-L2 434/Bu and cloned into pEGFP-N1 and/or pmCherry–C1 vectors, in order to obtain N-terminal Cherry-tagged and/or C-terminal EGFP-tagged fusions, as specified. For transfections, approximately 5x10^4^ HeLa cells/well were seeded onto glass coverslips placed in a 24-well plate the day before the experiment. The following day, cells were transfected with different constructs using jetPRIME (Polyplus-transfection Inc., NY, USA) according to manufacturer’s instructions. The second day, transfected cells were treated with oleic acid 200 μM for 8 hours before fixation.

### Neutral lipid staining, indirect immunofluorescence (IF) microscopy and fluorescence-based quantification of LDs

For neutral lipid staining in the absence of immunostaining, cells in coverslips were first washed with PBS twice, fixed with 3% formaldehyde/ 0.025% glutaraldehyde at room temperature for 20 min and stained with BODIPY 493/503 (Molecular Probes, Invitrogen Life Technologies, Carlsbad, California, USA) according to manufacturer’s instructions. For neutral lipid staining of isolated LDs, a saturated solution of BODIPY 493/503 in DMSO was diluted 1:1,000 in a suspension of purified LDs in PBS, incubated for 10 min at room temperature and then 3 μl of the stained suspension was transferred to glass microscopy slides and coverslips for visualization. For indirect immunofluorescence (IF) of LD-associated proteins, cells were fixed as described above, then permeabilized and blocked with 0.05% saponin and 2% BSA in PBS (SBP) for 30 min at room temperature followed by incubation with the specified primary antibodies for LD-associated proteins for 1 h on ice. Cells were then washed three times with SBP and incubated with either Alexa Fluor-conjugated anti-mouse or anti-rabbit IgG secondary antibodies (Invitrogen) for 30 min on ice. For neutral lipid staining in IF experiments, BODIPY 493/503 was used after incubation with the secondary antibodies, when indicated. For IF staining of endogenous inclusion membrane proteins, around 2x10^4^ HeLa cells/well were seeded onto glass coverslips the day before the experiment. Next, cells were infected with *C*. *trachomatis* LGV-L2 434/Bu (MOI < 1). At 26 hpi, cells were stimulated with oleic acid (100 μM) for additional 14 hours and then fixed with 3% formaldehyde/0.025% glutaraldehyde at room temperature for 20 min and permeabilized with triton 0.1% in PBS for 15 min at room temperature. Primary antibodies used for staining of inclusion membrane proteins were anti-Cap1 (rabbit polyclonal, kindly provided by A. Subtil, Pasteur Institute), anti-CTL0882 (CT618, rabbit polyclonal, generated in our laboratory as previously described [39]) and anti-IncG (kind gift from T. Hackstadt, RML). Staining was carried out as described above except that for samples stained with anti-CTL0882 (CT618), a one minute methanol treatment on ice was performed after fixation with 3% formaldehyde/0.025% glutaraldehyde. DNA was stained with Hoechst 33342 (Molecular Probes, Invitrogen Life Technologies, Carlsbad, California, USA) following manufacturer’s protocol. In all microscopy experiments, cells were mounted on microscope slides with Mowiol (Calbiochem, Germany). Epifluorescence microscopy images were acquired with a Zeiss Axioscope microscope equipped with a Hamimatsu CCD camera (Carl Zeiss, Germany) and processed with Axiovision v3.0 imaging software. Confocal images were acquired using a Zeiss LSM 510 inverted confocal microscope (Carl Zeiss, Germany). For fluorescence microscopy-based quantification of LDs, a previously described method was used [40], with few modifications. Briefly, HeLa cells infected or not for the indicated times, treated or not with OA at the indicated concentrations for 14 h were fixed with 3% formaldehyde/0.025% glutaraldehyde, stained with BODIPY 493/503-wihtout permeabilization, and mounted on Mowiol as described above. Epifluorescence images in the green channel were opened in TIFF format and analyzed using MBF ImageJ software (developed by Wayne Rasband, National Institutes of Health, Bethesda, MD; available at http://rsb.info.nih.gov/ij/index.html). Regions of interest were defined outlining individual cells, images were inverted and thresholds were applied by default settings. BODIPY-positive particles were counted, the total area of BODIPY-positive structures was annotated and the total area of BODIPY-positive elements per cell was calculated. At least 25 cells per each experimental condition were analyzed in three independent experiments. For fluorescence microscopy-based quantification of LDs, all images were taken with the same instrument setting and analyzed with the same ImageJ settings.

### Transmission electron microscopy

For transmission electron microscopy (TEM), cells were fixed in the presence of malachite green using a protocol specifically designed to preserve lipid-rich structures [41] with minor modifications. Briefly, cells were fixed for 2h at room temperature with 2.5% glutaraldehyde and 0.05% malachite green in 0.1 M sodium cacodylate buffer (pH 6.8). Post-fixation was carried out for 30 min with 0.5% osmium tetroxide and 0.8% potassium ferricyanide in 0.1 M sodium cacodylate, followed by 1 h in 1% tannic acid and 1 h in 1% uranyl acetate at room temperature. To visualize isolated LDs by TEM, a drop of the LD-enriched fraction was placed on a Formvar-covered, carbon-coated nickel grid, fixed/post-fixed as described above, mixed with collagen (Vitrogen) and solidified at 37°C as previously described [42]. Samples were dehydrated with a graded ethanol series and embedded in Spurr’s resin. Ultrathin sections were processed, post-stained with uranyl acetate and lead citrate, and imaged on a Tecnai G2 Twin microscope (FEI).

### IFU assays

To determine the number of inclusion forming units (IFU), confluent wild type MEFs, *dgat1/2* double knock-out, DGAT1-complemented and control *dgat1/2* cells monolayers were infected with *C*. *trachomatis* LGV-L2 434/Bu at an MOI ~0.5 as described above in two sets of 96 well plates (one for input and one for output IFUs) in triplicates. At 30 hpi, cells were either fixed with 100% methanol (EMD Millipore) for 10 min on ice (input plate) or lysed by hypotonic lysis with sterile water (output plate), followed by addition of 5xSPG to a final concentration of 1xSPG (200 μl per well) and stored at -80°C, as previously described [43]. For enumeration of output IFUs, confluent HeLa cell monolayers were infected by centrifugation at 3,000x*g* for 25 min at 10 °C (as described above) with serial dilutions of the lysates obtained from the output plates, incubated for 30 h and methanol-fixed. Inclusions from input and output plates were immunostained with polyclonal anti-LGV-L2 sera followed by Alexafluor-conjugated secondary antibodies as described previously [44]. Inclusions were counted using a Cellomics ArrayScan Vti HCS automated fluorescent imaging system (ThermoFisher).

### Mass spectrometry and data analysis

One microgram of peptide digest from each of the eight samples (20 hour and 40 hour, infected and uninfected in biological duplicate) was analyzed three times each (totaling six determinations per experimental condition) using a nanoAcquity UPLC system coupled to a Synapt HDMS mass spectrometer (Waters Corp, Milford, MA). The peptide sample was first trapped for 5 minutes at 5 μl/min at 99.9/0.1 v/v water/acetonitrile (0.1% formic acid) on a 5 μm Symmetry C18 180 μm × 20 mm trapping column. Peptide separations were performed over 120 minutes using a gradient of 5 to 40% acetonitrile (0.1% formic acid) and a flow rate of 0.3 μl/min, at 45°C column temperature on a 1.7 μm Acquity BEH130 C18 75 μm × 250 mm column (Waters). We conducted three data-independent analyses (MS^E^) analysis and one data-dependent analysis (DDA) of each sample, for a total of 32 sample injections. Samples were analyzed separately for the 20 hour and 40 hour studies, with samples interwoven between infected and uninfected condition, enabling robust statistical comparison of infected versus uninfected condition at each timepoint, but not between timepoints. Data-independent analyses were used for peptide and protein quantification, with data acquired using the MS^E^ (all ions fragmentation) method, 0.9 sec cycle time alternating between low collision energy (6 V) and high collision energy ramp (15 to 40 V). The data-dependent analysis (DDA) mode utilized a 0.9 sec MS scan followed by MS/MS acquisition on the top 3 ions with charge greater than 1. MS/MS scans for each ion used an isolation window of approximately 3 Da, a maximum of 4 seconds per precursor, and dynamic exclusion for 120 seconds within 1.2 Da of the selected precursor.

Rosetta Elucidator v3.3 (Rosetta Biosoftware, Inc., Seattle, WA) was utilized for peak detection and label-free quantification, using default parameters with the following exceptions: lockmass correction at 785.8426 m/z, peak volume quantification, and minimum peak time score 0.5. Feature intensities for each injection were subjected to robust mean normalization (top and bottom 10% of features excluded) in order to correct for protein column loading variability and/or variation caused by instrument response drift. Protein quantities were estimated based on averaging the intensities of the top 3 most intense peptides for each protein, and calculating the ratio to the same average for a protein of known quantity (ADH1_YEAST), which was spiked in at a known quantity (25 or 50 fmol per injection). In this way, we made an estimation of the mol quantity of each protein in the lipid droplet under infected versus uninfected condition, which could be compared based on a fold-change or statistical calculation such as a t-test.

We utilized both DDA and MS^E^ to generate peptide identifications. For DDA acquisition files, *.mgf searchable files were produced in Rosetta Elucidator and searches were then submitted to and retrieved from the Mascot v2.2 (Matrix Sciences, Inc) search engine in an automated fashion. For MS^E^ data, ProteinLynx Global Server 2.4 (Waters Corporation) was used to generate searchable files which were then submitted to the IdentityE search engine (Waters Corporation, Milford, MA). Precursor ion mass tolerance was 20 ppm for both PLGS and Mascot searches, and product ion tolerance was 0.04 Da for Mascot and 40 ppm for PLGS, and full tryptic specificity was used. A maximum of 2 missed cleavages were allowed. Carbamidomethyl cysteine was included as a fixed modification, and deamidation (NQ) and oxidation (M) were allowed as dynamic amino acid modifications. To enable global spectra scoring across results from both search engines, all search results were concurrently validated using the PeptideProphet and ProteinProphet algorithms in Elucidator using independent reverse decoy database validation, and were curated to a 1% FDR. The entire raw data expression at the peptide and protein level, for all samples, prior to quantitative analysis is available on S1 Table.

### Protein grouping and analysis of quantitative changes in LD proteome

In order to analyze quantitative changes in the LD proteome in infected vs. uninfected cells, only proteins detected with at least 3 peptides to match and with highly reproducible quantitative data [within group coefficient of variation (%CV) < 25% across the replicates] were considered (S2 and S3 Tables). The resulting final list of host LD-proteins (n = 107) was uploaded and then retrieved from UniProt-KB (http://www.uniprot.org/) for browsing by gene ontology (GO). The quantitative changes in GO-based protein groups and subgroups were then analyzed. Individual proteins for which the fold change compared to uninfected controls was > 1.5 or < -1.5 (*p* < 0.05, T-test, two-tailed, paired) were considered significantly changed. *Chlamydia* proteins, present only in infected samples as expected, were analyzed separately.

## Results and Discussion

### Isolation and purification of LDs

**Fig 1:**
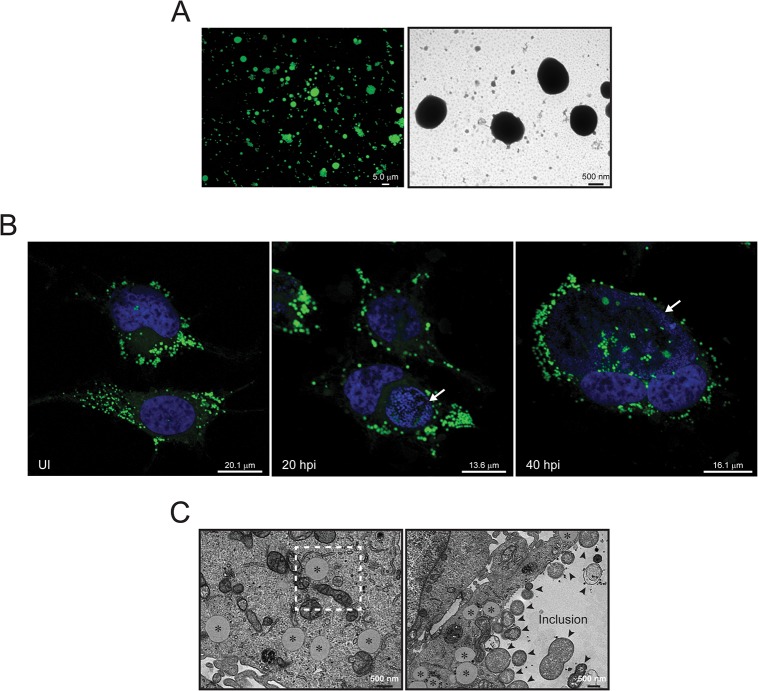
Morphology of LDs in uninfected and *C*. *trachomatis*-infected HeLa cells. (A) A fresh suspension of LDs isolated from HeLa cells was stained with BODIPY 493/503 for 15 min and imaged by fluorescence microscopy (left panel) or processed for TEM (right panel). LDs appear as intact spherical structures. Since no morphological differences between LDs from uninfected and infected cells were observed, a representative image of LDs isolated from uninfected cells is shown (B) Representative confocal images of HeLa cells, uninfected (UI) or infected with *C*. *trachomatis* LGV-L2 for 20 h (20 hpi) or 40 h (40 hpi). Cells were stained with BODIPY 493/503 to visualize LDs (green) and Hoechst was utilized to visualize host and bacterial DNA (blue). Bacterial replicative vacuoles (inclusions) are indicated with white arrows. (C) TEM images of uninfected (left panel) and *C*. *trachomatis* infected (20 hpi, right panel) HeLa cells. LDs (asterisk) and *C*. *trachomatis* (black arrows) are indicated. Note the close apposition of LDs with mitochondria and ER-like membranes (dashed square, left panel) and with the inclusion (right panel). In all cases (A-C) HeLa cells were stimulated with oleic acid 100 μM for 14 h prior to fixation and/or staining to enhance LD production.

We prepared highly purified LDs from HeLa cells, a human cervical adenocarcinoma epithelial cell line that is widely used to study *C*. *trachomatis* biology. To increase the yield of LDs for proteomics analysis, we stimulated LD formation by supplementing the growth media with 100 μM oleate 14 hours prior to organelle harvesting. We first assessed the enrichment of LDs in fractions isolated after two sequential density gradient ultracentrifugation steps by comparing the total protein composition of LD fractions to that of total lysates. Proteins extracted from highly enriched LD fractions displayed a relatively simple band pattern compared to that of total cell lysates (S1 Fig). We then tested the level of cross contamination of the LD samples with other organelles by immunoblot analysis with a panel of antibodies against protein markers for specific cellular compartments. Compared to total lysates, the LD fraction was highly enriched in resident LD proteins, like the perilipin family proteins PLIN2 (previously ADRP) and PLIN3 (previously Tip47), the acyl-CoA synthases ACSL-3 and ACSL-4, NSDHL (sterol-4-alpha-carboxylate 3-dehydrogenase, decarboxylating), PLPL2 (patatin-like phospholipase domain-containing protein 2, also known as adipose triglyceride lipase, ATGL), Aup1 (ancient ubiquitous protein 1) and FAF2 (FAS-associated factor 2, also known as UBX domain-containing protein 8, UBXD8) (S1 Fig). In contrast, the ER marker TRAP-α was barely detected in the LD fraction, and the plasma membrane marker integrin-α2 as well as the cytosolic marker GAPDH (glyceraldehyde-3-phosphate dehydrogenase) were clearly depleted from LD preparations. We also assessed the quality of the LD preparations by fluorescence microscopy. A fresh suspension of the separated fraction of LDs showed abundant and apparently intact BODIPY 493/503-stained spherical structures (Fig 1A, left panel). Most LDs varied in sizes ranging from ~1–5 μm and sometimes appeared to form clusters. To further assess the morphology as well as the purity of the LD preparations, we performed TEM on the isolated LDs. Consistent with the fluorescence images, LDs appeared as very small, seemingly intact spherical structures (Fig 1A, right panel). No other associated membranous structures were observed. Next, in order to assess if any changes were induced in the LD proteome upon infection with *C*. *trachomatis*, LDs were isolated from uninfected cells and from cells infected with *C*. *trachomatis* LGV-L2 for 20 hours (mid-cycle) or 40 hours (late-cycle). Both uninfected and infected HeLa cells displayed abundant LDs (Fig 1B). We performed an ultrastructural analysis by transmission electron microscopy (TEM) of LDs in uninfected and *C*. *trachomatis*-infected cells fixed in the presence of malachite green to preserve lipid structures [41]. Cytoplasmic LDs were readily identifiable organelles with a thin membrane monolayer and a weakly stained, uniform core. In uninfected cells, LDs were frequently found closely apposed to mitochondria and ER-like membranes (Fig 1C, left panel), as previously reported [45, 46]. In infected cells, LDs were also found frequently associated to the inclusion membrane (Fig 1C, right panel) [28, 30].

### Label free quantification of the LD proteome

The proteins extracted from LDs were digested with trypsin and analyzed by LC-MS/MS, allowing for the application of label-free, MS-based quantification of proteins as previously described [34]. The entire raw data expression at the peptide and protein level for all samples, prior to quantitative analysis, shows a total of 257 identified proteins (S1 Table). Only proteins identified by at least 3 unique peptides and present across all 8 independent LD samples, were considered for quantification. We excluded from our quantitative analysis, proteins for which the “within group coefficient of variation” (%CV) of the quantitative determination across the replicates was greater than 25% in at least one of the experimental conditions. This analysis led to a compendium of 107 human proteins identified and quantified in all LD samples. Importantly, among these 107 host proteins, 85 (~80%) were previously reported as LD-associated proteins (Table 1) further supporting the purity of the LD-fractions analyzed. All MS-based quantitative data is available in S1–S4 Tables.

**Table 1:** List of proteins represented abundantly in LD extracts from uninfected and *C*. *trachomatis*-infected HeLa cells.

Protein group	Protein ID			Uniprot	Protein description	Peptides to match	Previous reports	
Perilipins and lipid metabolism	Q99541			PLIN2_HUMAN	perilipin-2	38	[47–49]	
	O60664			PLIN3_HUMAN	perilipin-3	97	[47, 50]	
	Q8WTS1			ABHD5_HUMAN	1-acylglycerol-3-phosphate o-acyltransferase ABDH5	21	[51]	
	O95573			ACSL3_HUMAN	long-chain-fatty-acid-ligase 3	73	[48, 51]	
	O60488			ACSL4_HUMAN	long-chain-fatty-acid-ligase 4	25	[48]	
	P25705			ATPA_HUMAN	ATP H^+^ mitochondrial f1 alpha subunit	13	[49, 52, 53]	
	P06576			ATPB_HUMAN	ATP synthase subunit mitochondrial	12	[47, 49, 52–56]	
	Q8NBQ5			DHB11_HUMAN	estradiol 17-beta-dehydrogenase 11	35	[57]	
	P56937			DHB7_HUMAN	hydroxysteroid (17-beta) dehydrogenase 7	9	[42, 47, 58]	
	O75911			DHRS3_HUMAN	short-chain dehydrogenase reductase 3	6	[55]	
	P30084			ECHM_HUMAN	enoyl-CoA hydratase, mitochondrial	3	[46, 53, 55]	
	P48449			ERG7_HUMAN	lanosterol synthase	53	[48, 51]	
	Q643R3			LPCT4_HUMAN	lysophospholipid acyltransferase LPCAT4	5	[58]	
	Q15738			NSDHL_HUMAN	sterol-4-alpha-carboxylate 3-dehydrogenase, decarboxylating	44	[48, 51]	
	Q8NF37			PCAT1_HUMAN	lysophosphatidylcholine acyltransferase 1	40	[51]	
	Q7L5N7			PCAT2_HUMAN	lysophosphatidylcholine acyltransferase 2	9	[51]	
	Q96AD5			PLPL2_HUMAN	patatin-like phospholipase domain-containing protein 2	23	[51]	
	Q8IZV5			RDH10_HUMAN	retinol dehydrogenase 10	14	[57, 59]	
Other LD-associated proteins	Q9Y679			AUP1_HUMAN	ancient ubiquitous protein 1	32	[60]	
	Q07065			CKAP4_HUMAN	cytoskeleton-associated protein 4	3	[48]	
	Q96CS3			FAF2_HUMAN	FAS-associated factor 2	31	[48]	
	Q9Y5L2			HLPDA_HUMAN	hypoxia-inducible lipid droplet-associated protein isoform 1	5	[61]	
	Q9H8H3			MET7A_HUMAN	methyltransferase-like protein 7A	10	[57, 58]	
	Q8NBX0			SCPDL_HUMAN	saccharopine dehydrogenase-like oxidoreductase	32	[48]	
Small GTPases	P62820			RAB1A_HUMAN	ras-related protein Rab-1a	14	[46, 57]	
	P61019			RAB2A_HUMAN	ras-related protein Rab-2a	10	[46, 58]	
	Q8WUD1			RAB2B_HUMAN	ras-related protein Rab-2b	13	[46]	
	Q96E17			RAB3C_HUMAN	ras-related protein Rab-3c	4		
	O95716			RAB3D_HUMAN	ras-related protein Rab-3d	5		
	P20338			RAB4A_HUMAN	ras-related protein Rab-4a	3	[46]	
	P61018			RAB4B_HUMAN	ras-related protein Rab-4b	3	[46]	
	P51149			RAB7A_HUMAN	ras-related protein Rab-7a	26	[57, 58]	
	P61006			RAB8A_HUMAN	ras-related protein Rab-8a	12	[46, 49]	
	P61026			RAB10_HUMAN	ras-related protein Rab-10	14	[42, 46, 57]	
	Q15907			RB11B_HUMAN	ras-related protein Rab-11b	9	[42, 46, 62]	
	P61106			RAB14_HUMAN	ras-related protein Rab-14	17	[46, 47, 57, 63]	
Small GTPases	P51159			RB27A_HUMAN	ras-related protein Rab-27a	3		
	Q13636			RAB31_HUMAN	ras-related protein Rab-31	5	[46]	
	Q13637			RAB32_HUMAN	ras-related protein Rab-32	4		
	Q9BZG1			RAB34_HUMAN	ras-related protein Rab-34	5	[46]	
	Q15286			RAB35_HUMAN	ras-related protein Rab-35	5	[46]	
	P62826			RAN_HUMAN	GTP-binding nuclear protein Ran	4	[54]	
	P61224			RAP1B_HUMAN	ras-related protein Rap-1b	9	[46, 48, 58]	
Protein folding & heat-shock proteins	P27797			CALR_HUMAN	calreticulin	20	[53, 64, 65]	
	P27824			CALX_HUMAN	calnexin	6	[46, 47, 53, 55, 57, 65, 66]	
	P10809			CH60_HUMAN	heat shock 60kda protein 1	40	[46, 49, 53, 67]	
	P14625			ENPL_HUMAN	endoplasmin	15	[49, 57]	
	Q14697			GANAB_HUMAN	neutral alpha-glucosidase AB isoform 1	7	[55]	
	P38646			GRP75_HUMAN	stress-70 protein-mitochondrial	13	[55]	
	P34931			HS71L_HUMAN	heat shock 70 kDa protein 1-like	20	[46]	
	P07900			HS90A_HUMAN	heat shock protein HSP 90-alpha	6	[53]	
	P08238			HS90B_HUMAN	heat shock protein HSP 90-beta	14	[46, 68]	
	P08107			HSP71_HUMAN	heat shock 70 kDa protein 1A	7		
	P54652			HSP72_HUMAN	heat shock-related 70 kDa protein 2	13	[46]	
	P11142			HSP7C_HUMAN	heat shock 70 kDa protein 8	17	[53, 55, 64, 67]	
	P04792			HSPB1_HUMAN	heat shock protein beta-1	8	[57]	
	P07237			PDIA1_HUMAN	protein disulfide-isomerase precursor	19	[53, 55, 64, 67]	
	P30101			PDIA3_HUMAN	protein disulfide-isomerase A3 precursor	27	[55, 57]	
	Q15084			PDIA6_HUMAN	protein disulfide-isomerase A6 precursor	6	[42, 53, 55, 57]	
	P62937			PPIA_HUMAN	peptidyl-prolyl cis-trans isomerase A	5		
Cytoskeleton	P21333			FLNA_HUMAN	filamin-A	26	[65]	
	Q04695			K1C17_HUMAN	keratin, type I cytoskeletal 17	16		
	P05783			K1C18_HUMAN	keratin, type I cytoskeletal 18	11	[66]	
	P08729			K2C7_HUMAN	keratin, type II cytoskeletal 7	16		
	P05787			K2C8_HUMAN	keratin, type II cytoskeletal 8	24	[53, 66, 67]	
	P02545			LMNA_HUMAN	prelamin-A/C	15	[54]	
	Q15149			PLEC_HUMAN	low quality protein: plectin	13	[65, 69]	
	P68366			TBA4A_HUMAN	tubulin alpha-4a chain	15	[46, 57, 64]	
	Q13509			TBB3_HUMAN	tubulin beta-3 chain	22	[47, 64, 69]	
	P68371			TBB4B_HUMAN	tubulin beta-4B chain	12	[47, 64, 69, 70]	
	P08670			VIME_HUMAN	vimentin	30	[47, 49, 53]	
Glycolysis & Energy generation		P05141	ADT2_HUMAN		ADP/ ATP translocase 2	4	[53, 58, 65]	
		P06733	ENOA_HUMAN		alpha-enolase	12	[49, 53, 55, 65, 71]	
		P13804	ETFA_HUMAN		electron transfer flavoprotein subunit mitochondrial	3	[53]	
		P04406	G3P_HUMAN		glyceraldehyde-3-phosphate dehydrogenase	16	[48]	
		P62873	GBB1_HUMAN		guanine nucleotide-binding protein G(I)/G(S)/G(T) subunit beta-1	6	[46, 69]	
		P00338	LDHA_HUMAN		L-lactate dehydrogenase A chain	5	[49]	
		P07195	LDHB_HUMAN		L-lactate dehydrogenase B chain	7		
Translation		Q5VTE0	EF1A3_HUMAN		eukaryotic translation elongation factor 1 alpha 1	7		
		P13639	EF2_HUMAN		elongation factor 2	5		
		P49411	EFTU_HUMAN		elongation factor Tu, mitochondrial	6		
		Q8NHW5	RLA0L_HUMAN		60S acidic ribosomal protein P0-like	3		
Miscellaneousenzymes		P05023	AT1A1_HUMAN		Sodium/potassium-transporting ATPase subunit alpha-1	13	[55, 57]	
		Q96LJ7	DHRS1_HUMAN		dehydrogenase reductase sdr family member 1	42	[53, 55]	
		Q9BUP3	HTAI2_HUMAN		oxidoreductase HTATIP2	11		
		Q86SQ9	DHDDS_HUMAN		dehydrodolichyl diphosphate synthase	6	[72]	
		Q96E22	NGBR_HUMAN		Nogo-B receptor	12		
		P78527	PRKDC_HUMAN		DNA-dependent protein kinase catalytic subunit	6		
Other proteins		P62258	1433E_HUMAN		14-3-3 protein epsilon	4	[53]	
		P61981	1433G_HUMAN		14-3-3 protein gamma	3	[53]	
		P63104	1433Z_HUMAN		14-3-3 protein zeta delta	5	[69]	
		P80723	BASP1_HUMAN		brain acid soluble protein 1	29	[49]	
		P16070	CD44_HUMAN		CD44 antigen	3		
		P13987	CD59_HUMAN		CD59 glycoprotein	7		
		Q8IZ81	ELMD2_HUMAN		ELMO domain-containing protein 2	4	[72]	
		Q8N128	F177A_HUMAN		Protein FAM177A1	3		
		P11021	GRP78_HUMAN		78 kDa glucose-regulated protein	35	[55, 57]	
		Q14974	IMB1_HUMAN		karyopherin beta 1	4	[65]	
		Q99623	PHB2_HUMAN		prohibitin 2	3	[47, 49, 53, 58]	
		Q6S8J3	POTEE_HUMAN		POTE ankyrin domain family member E	5	[66]	
		P06454	PTMA_HUMAN		prothymosin alpha	3		
		P50454	SERPH_HUMAN		serpin H1	5	[46, 53, 55]	
		Q01105	SET_HUMAN		protein SET	3		
		P55327	TPD52_HUMAN		tumor protein D52	6		
		Q92575	UBXN4_HUMAN		UBX domain-containing protein 4	5	[70]	

This table shows all proteins identified by LC-MS/MS in all samples (8 independent LD purifications, totaling 24 mass spectrometry determinations) with at least 3 unique peptides and with high reproducibility in the quantitative determination across the replicates (within group %CV less than 25% across 6 replicates for each experimental condition). Protein groups, identification, names and description according to UniProtKB, as well as number of unique peptides detected per protein are specified. The “Previous reports” column highlights proteins previously identified in LD fractions.

**Fig 2:**
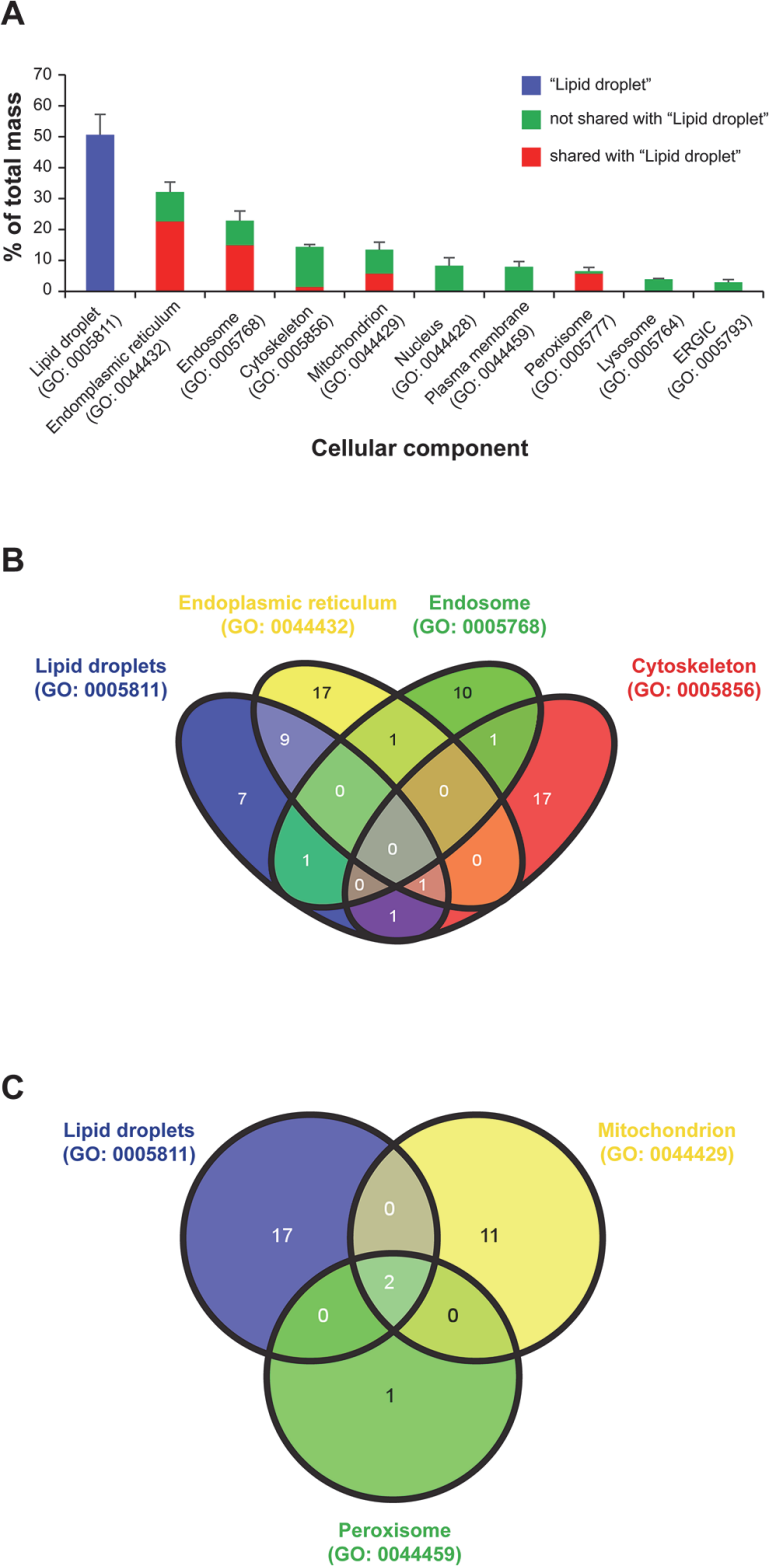
Cellular component analysis of LD-associated proteins. **(A)** All LD-associated proteins identified and quantified in this study were classified based on their gene ontologies (GO) by “cellular component” using UniProtKB. The quantitative contribution of each protein group (average across all samples) was calculated and expressed as percent of the total mass of LD-associated proteins. Only the quantitatively more abundant categories are represented (see S5 Table for a detailed list). Because a given protein can be classified in more than one GO category, there is some degree of overlap between the groups and thereby the sum of all categories exceeds one hundred percent. The quantitative contribution of proteins belonging to the “lipid droplet” category (GO: 0005811) as well as that corresponding to proteins “shared” and “not shared” between the “lipid droplet” and the other specified categories are indicated. **(B, C)** Venn diagrams show the number of overlapping proteins between “lipid droplets” and other selected cellular components.

To explore the potential function(s) of LDs during infection, we classified the quantified LD proteins based on gene ontologies (GO) (S5 Table) and analyzed the contribution of specific protein groups to the total mass. Not surprisingly, about 50% of the mass corresponded to proteins classified within the cellular component “lipid droplet” (GO: 0005811) (Fig 2A). The LD structural protein PLIN3 alone, was by far the most abundant protein (S4 Table) representing 15% of the total mass (average across all samples). Upon ranking all proteins by their relative abundance, 9 out of the 10 most abundant proteins represented known LD proteins (S4 Table and Table 1) and accounted for 44% of the total mass (average across all samples). Additional abundant protein groups corresponded to ER (GO: 0044432) and endosome (GO: 0005768) categories, representing about 30% and 20% of the total mass, respectively (Fig 2A), although the bulk of the mass corresponding to these categories was due to proteins shared with the “lipid droplet” (GO: 0005811) category (Fig 2A and 2B). Cytoskeletal (GO: 0005856) and mitochondrial (GO: 0044429) proteins were also abundant (~14% of the total mass), as were “peroxisome” (GO: 0044459) (Fig 2A–2C). Overall, these results confirm that our LD samples were highly enriched in known LD-associated proteins and that contamination with other cellular compartments was minimal.

### Quantitative changes induced in the LD proteome in response to infection with *C*. *trachomatis*

**Fig 3:**
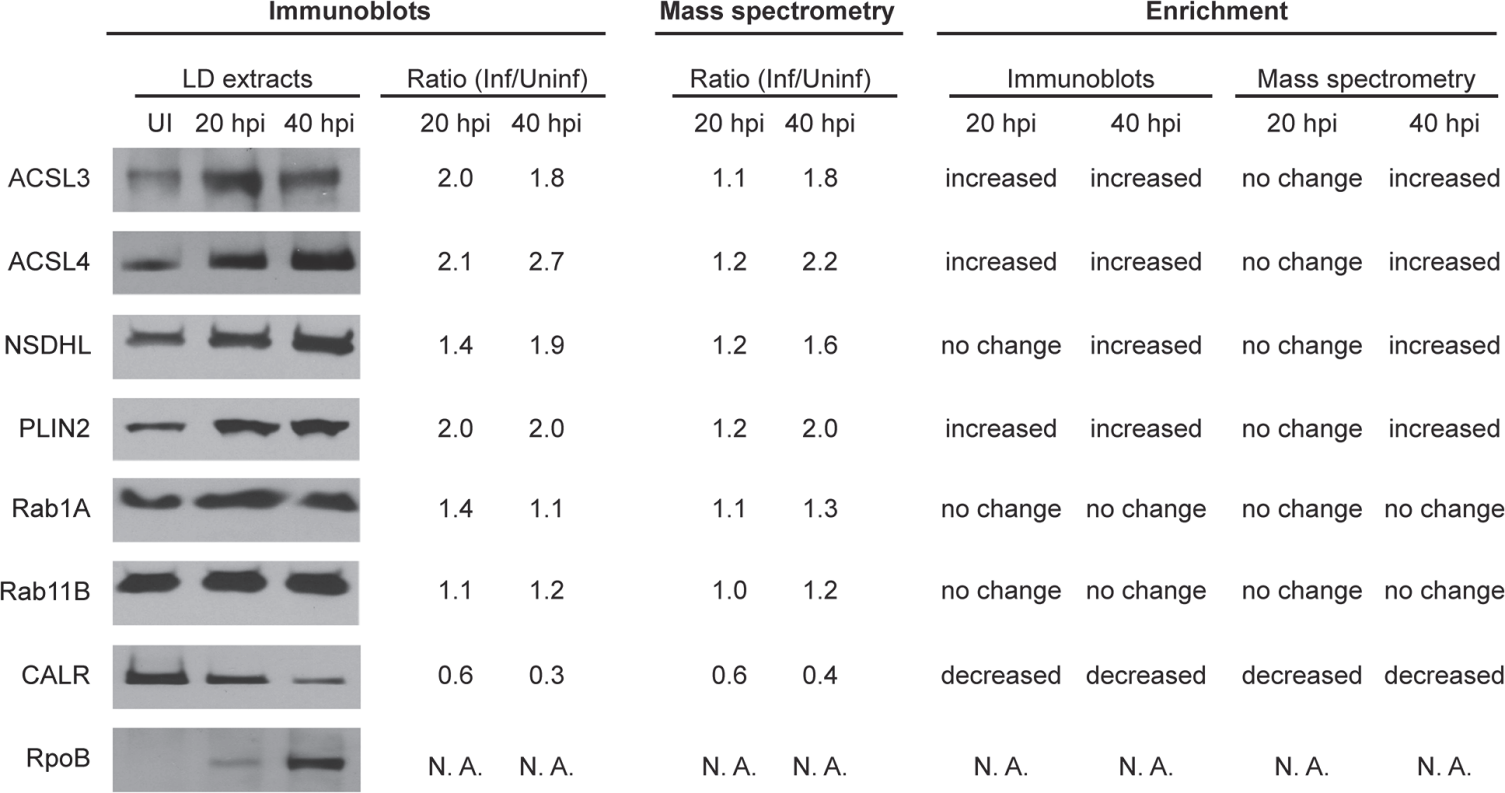
Immunoblot analysis of selected proteins identified in LD-extracts by proteomics. LD protein extracts were obtained from HeLa cells uninfected (UI) or *C*. *trachomatis*-infected for 20 h (20 hpi) and 40 h (40 hpi). Protein content was assessed by Bradford assay, equal amounts per lane were separated by SDS-PAGE and transferred to nitrocellulose membranes. The relative abundance of a subset of proteins associated to LD-extracts was assessed by immunoblot densitometry analysis (using ImageJ software) and compared with quantitative changes obtained from mass spectrometry (MS)-based quantification (expressed as the ratio, infected/uninfected). Proteins were considered “increased” or “decreased” when the Inf/Uninf ratio was > 1.5 or < 0.67 (this ratio representing a fold change greater or equal to -1.5), respectively, as shown in the “Enrichment” columns. Host proteins assessed were: ACSL3 (long-chain-fatty-acid-ligase 3); ACSL4 (long-chain-fatty-acid-ligase 4); NSDHL (sterol-4-alpha-carboxylate 3-decarboxylating); PLIN2 (perilpin-2); Rab1 (ras-related protein Rab-1a); Rab11 (ras-related protein Rab-11b); CALR (calreticulin). *C*. *trachomatis* RpoB (RNA polymerase subunit beta) was used as a marker of infection. A representative image is shown. N.A., “not applicable”.

**Fig 4:**
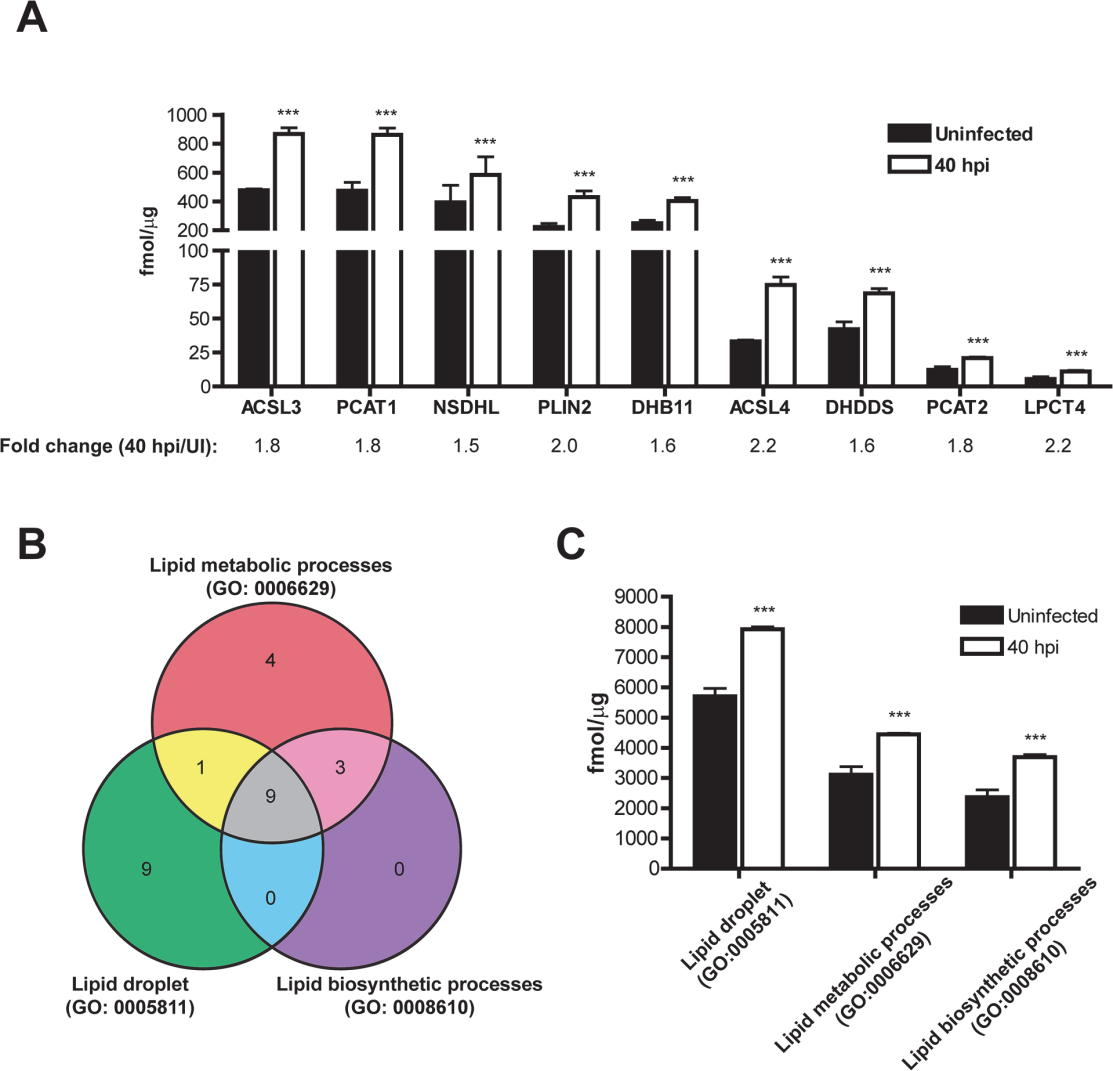
LDs isolated from *C*. *trachomatis-*infected cells are enriched in proteins related to lipid metabolism. **(A)** The expression levels of all proteins belonging to “lipid metabolic processes” (GO: 0006629) increased in LD fractions at 40 hpi are represented. Bars represent the mean (fmol μg^-1^) resulting from six mass spectrometry-based determinations (as detailed in supporting information S3 Table). Error bars indicate the standard deviation. Asterisks (***) indicate statistically significant differences (*p* < 0.001, two-tailed T-test). Fold changes (40 hpi/Uninfected, average across 6 replicates) are indicated. **(B)** Venn diagram summarizing the overlap between the protein groups “Lipid metabolic processes” (GO: 0006629), “Lipid droplet” (GO: 0005811) and “Lipid biosynthetic processes” (GO: 0008610) identified in this study (see S5 Table for a detailed list). **(C)** Proteins were grouped into the indicated GO categories and the total mass for each category was calculated. Bars represent the mean (fmol μg^-1^) resulting from six MS-based determinations. Error bars represent the standard deviation. Asterisks (***) indicate statistically significant differences (*p* < 0.001, two-tailed T-test). ACSL3 (long-chain-fatty-acid-ligase 3); PCAT1 (lysophosphatidylcholine acyltransferase 1); NSDHL (sterol-4-alpha-carboxylate 3-decarboxylating); PLIN2 (perilpin-2); DHB11 (estradiol 17-beta-dehydrogenase 11); ACSL4 (long-chain-fatty-acid-ligase 4); DHDDS (dehydrodolichyl diphosphate synthase); PCAT2 (lysophosphatidylcholine acyltransferase 2); LPCT4 (lysophospholipid acyltransferase LPCAT4).

To assess the impact of *C*. *trachomatis* infections to the function of LDs, we analyzed the changes in the LD proteome at 20 hpi (mid-cycle) and 40 hpi (late-cycle). We considered those LD proteins that changed > 1.5 or < -1.5 fold in abundance compared to uninfected controls with a *p* value < 0.05 (S6 Table). Very few statistically significant changes were observed at 20 hpi. In contrast, we found that at 40 hpi, 64 proteins (60% of the total) were differentially expressed, including 23 proteins whose abundance increased in LDs, and 41 proteins whose abundance decreased (S6 Table). Therefore, our analysis is centered on the 40 hpi time-point. To validate the MS-derived quantitative data, we selected a number of proteins predicted to be increased, decreased or unchanged and performed western blot analysis. As shown in Fig 3, the trends in protein abundance on LDs as determined by MS-based quantification were similar to those observed by immunoblot. The 23 proteins increased in LDs of cells infected for 40 h (S3 and S6 Tables), most of which were abundant proteins, accounted for 37% of the total mass of the LD and included several proteins implicated in functions related to lipid metabolism and storage (Fig 4A), one protein involved in lipid particle organization (Rab-18) and one protein associated with intracellular cholesterol transport (Nogo B receptor) (S6 Table). Indeed, by gene ontology, the mass of proteins belonging to categories like “Lipid metabolic processes”, “Lipid droplet” and “Lipid biosynthetic processes” (Fig 4B) were found to be significantly increased at this time point (Fig 4C). Interestingly, when analyzing the overlap between these three GO categories (Fig 4B), we found that 12 out of 17 proteins detected in LD-fractions and classified within “Lipid metabolic processes” were involved in lipid biosynthesis. Moreover, out of 9 proteins increased at 40 hpi functionally classified within “Lipid metabolic processes”, 8 were involved in lipid biosynthesis and 7 classified within the cellular component “Lipid droplet” (S6 Table). Overall these findings indicate that lipid droplet biogenesis is induced in *C*. *trachomatis*-infected cells.

When we carried out the gene ontology analysis of LD-associated proteins that were decreased at 40 hpi we found that “response to unfolded proteins” and “generation of precursor metabolites and energy” were over represented (S6 Table). This latter category was also reported as being destabilized in a Global Protein Stability (GPS) study of alterations induced by *C*. *trachomatis* infection [73]. The significance of these changes is unclear.

Overall, these results indicate that the protein composition of LDs is modified in response to *C*. *trachomatis*, particularly at late stages post-infection.

### *C*. *trachomatis* inclusion membrane proteins associate with LDs

**Fig 5:**
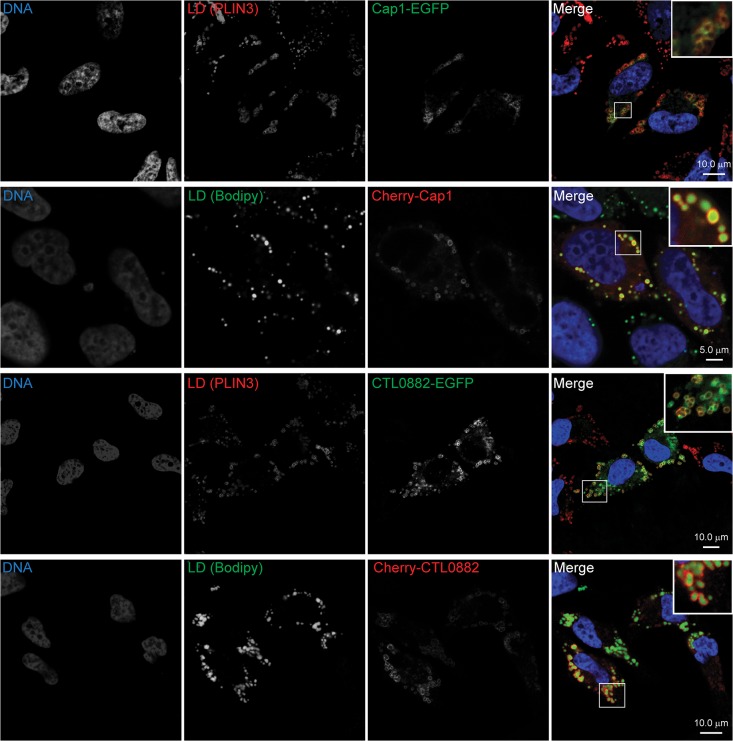
*C*. *trachomatis* inclusion membrane proteins Cap1 and CTL0882 co-localize with LDs. Representative confocal images of HeLa cells transfected with constructs encoding Cap1 or CTL0882 C-terminally fused to EGFP or N-terminally fused to Cherry, stimulated with oleic acid 200 μM for 8 h prior to fixation. LD labeling was done by either immunostaining of the LD structural protein PLIN3 (red channel) or by neutral lipid staining with BODIPY 493/503 (green channel). Hoechst staining was used to visualize DNA (blue). Bar sizes are shown in the merge panels.

**Fig 6:**
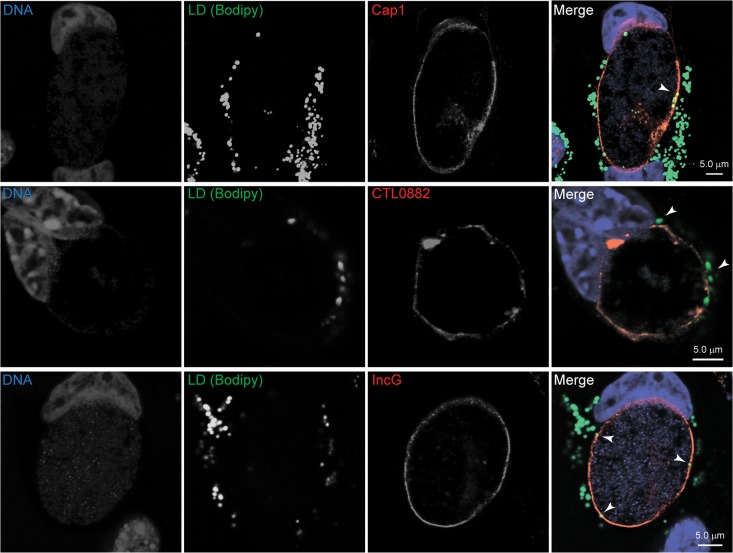
LDs associate with Cap1, CTL0882 and IncG at the inclusion membrane. Representative confocal images of HeLa cells infected with *C*. *trachomatis* (40 hpi), stimulated with oleic acid (100 μM, 14h prior to fixation). Immunofluorescence staining of Cap1, CTL0882 and IncG was carried out (red channel) and LDs were visualized using the neutral lipid-specific BODIPY 493/503 staining (green channel). Hoechst staining was used to visualize DNA (blue). Bar sizes are shown in the merge panels. Arrowheads highlight LDs found in close apposition with Cap1, CTL0882 and IncG in the inclusion membrane, as indicated.

*Chlamydia* proteins were also found associated with LDs. Out of a total of 6 bacterial proteins detected, only three were quantifiable. These included three inclusion membrane proteins (Incs): Cap1 (CTL0791/ CT529), CTL0882 (CT618) and IncG (CTL00373/CT118) (S7 Table). Incs are *Chlamydia*-specific, putative type III-secreted effectors that localize to the inclusion membrane through a bi-lobed hydrophobic motif [74]. More than 50 predicted Inc proteins are encoded in the *C*. *trachomatis* genome [75] and their functions are likely important for inclusion membrane biogenesis and structure [76]. We expressed these proteins ectopically in mammalian cells as fluorescently tagged fusion proteins. In the presence of oleic acid stimulation, N-terminally tagged Cherry and C-terminally tagged EGFP fusions of Cap1 and CTL0882 clearly co-localized with LDs (Fig 5). For ectopically expressed Cherry-IncG, however, we found association with LDs only occasionally and the protein mainly displayed a reticular localization pattern (S3 Fig). Localization of all these inclusion membrane proteins when ectopically expressed in the absence of oleic acid stimulation was either punctate or reticular, as detailed in S2 and S3 Fig. In order to assess the localization of endogenous Cap1, CTL0882 and IncG, we infected HeLa cells with *C*. *trachomatis* (40 hpi) and stimulated LD production with oleic acid (100 μM, 14h prior to fixation). We then carried out immunofluorescence microscopy with Cap1, CTL0882 or IncG antibodies and stained LDs with BODIPY 493/503. As expected, Cap1, CTL0882 and IncG were present in the inclusion membrane (Fig 6). LDs were frequently observed closely apposed to Cap1, CTL0882 and IncG in the inclusion membrane, but we did not observe a preferential enrichment of these Inc at those attachment sites, at least at the resolution of our microscopy analysis. We note that IncG, Cap1 and CTL0882 are not abundant proteins in EBs or RBs [77], so their association with LD fractions indicates a marked enrichment over total protein levels. The expression of inclusion membrane proteins with affinity for LDs may represent a bacterial strategy to promote the previously reported close association of these organelles with inclusion membranes [28, 30, 78]. The other three additional chlamydial proteins detected were excluded from our analysis since they were identified with only 2 unique peptides. These were the major outer membrane protein MOMP, the virulence plasmid-encoded protein pGP3D (these two are likely contaminants, as they are among the most abundant proteins in *C*. *trachomatis* [77]) and the inclusion membrane protein CrpA. Previous work from our laboratory identified at least two chlamydial proteins, Lda1 and Lda3 [28], localized to LDs when ectopically expressed as EGFP fusions in HeLa cells. In our study, we did not detect endogenous Lda1 or Lda3 in LD fractions. However, we cannot discard the possibility that these-or other- chlamydial proteins went undetected due to their expression level being below the detection limit.

### The LD content increases in *C*. *trachomatis-*infected cells and impaired LD biogenesis negatively affects chlamydial replication

**Fig 7:**
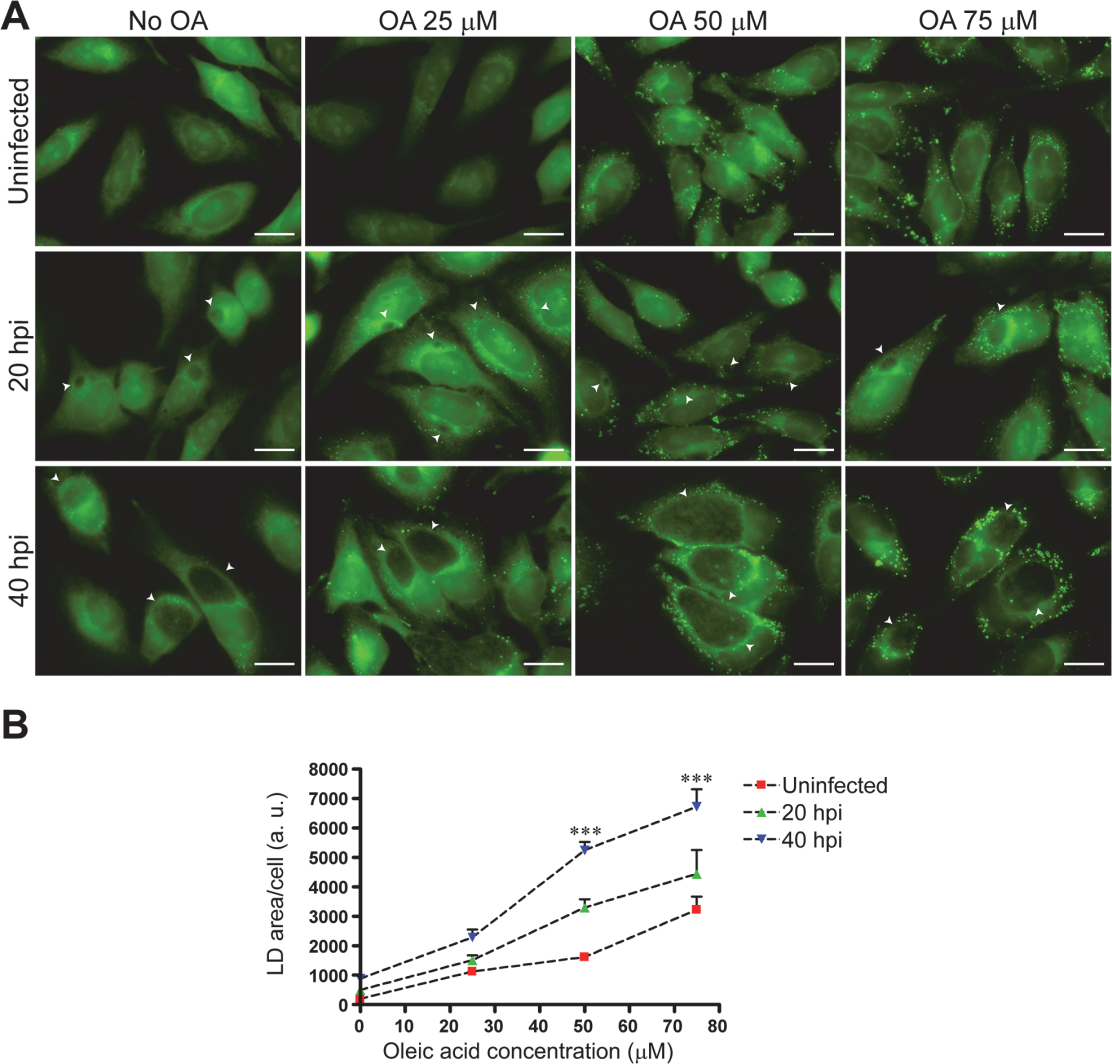
*C*. *trachomatis-*infected cells display increased amounts of LDs. **(A)** Representative fluorescence microscopy images of HeLa cells uninfected or infected with *C*. *trachomatis* (20 hpi or 40 hpi) and stimulated or not with the indicated micromolar concentrations of oleic acid (OA) for 14h prior to fixation, as specified. For visualization of LDs, neutral lipid-specific BODIPY 493/503 staining was used (green). Chlamydial inclusions are highlighted (white arrowheads). Bar sizes (20 μm) are indicated. **(B)** Fluorescence microscopy-based quantification of LD content was carried out using MBF-ImageJ software (as described in Materials and Methods) by measuring the total area of BODIPY 493/503 positive structures expressed as arbitrary units (a. u.) per cell. At least 30 cells per condition were analyzed in three independent experiments. Mean and SEM are represented. Asterisks indicate statistically significant differences (*p*<0.001) as determined by ANOVA-Bonferroni post-test.

The LD proteome in *C*. *trachomatis*-infected cells (40 hpi) is characterized by an increase in the relative representation of proteins involved in lipid biosynthesis as compared to other resident LD proteins. As a result, we expected an increase in the number of LDs as a consequence of infection as previously suggested by thin layer chromatography analysis of neutral lipids in *C*. *trachomatis*-infected cells [28]. To test this premise, we infected cells with *C*. *trachomatis* in the presence or absence of various concentrations of oleic acid and carried out a fluorescence microscopy-based quantification of BODIPY 493/503 positive structures (LDs) within cells. As shown in Fig 7, *C*. *trachomatis-*infected cells accumulated more LDs than uninfected at 40 hpi in HeLa cells stimulated with oleic acid concentrations > 50 μM. The same trend (although not statistically significant) was also observed at 20 hpi and for all oleic acid concentrations tested.

**Fig 8:**
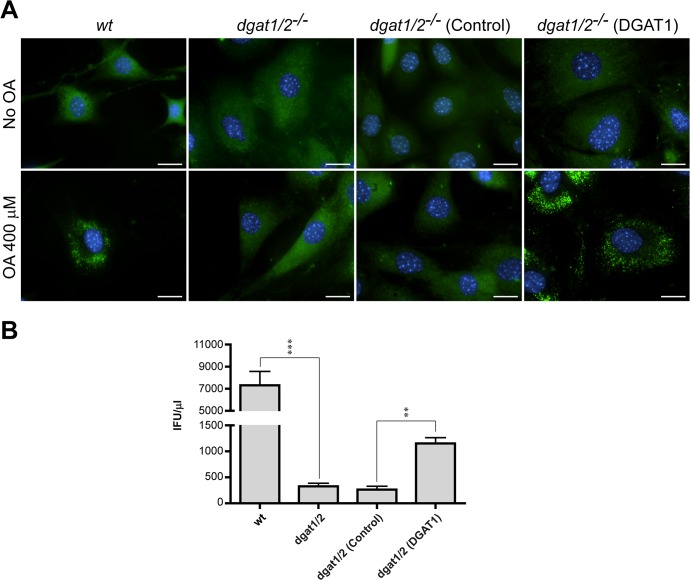
Production of *C*. *trachomatis* infectious progeny is impaired in cells devoid of LDs. **(A)** Representative fluorescence microscopy pictures of wild type MEF (*wt*), the diacylglycerol-acyltransferases 1 and 2 double knock-out (*dgat1/2* ^*-/-*^), *dgat1/2* ^*-/-*^ complemented with empty vector [*dgat1/2* ^*-/-*^ (Control)] and *dgat1/2* ^*-/-*^ complemented with DGAT1 [*dgat1/2* ^*-/-*^ (DGAT1)], stimulated or not with oleic acid (OA) 400 μM for 14 h, as indicated. For visualization of LDs, neutral lipid-specific BODIPY 493/503 staining was used (green). Hoechst staining was used to visualize DNA (blue). Bar sizes (20 μm) are shown. Note that *dgat1/2* ^*-/-*^ cells are devoid of LDs even under stimulation with OA, whereas LD formation is restored in *dgat1/2* ^*-/-*^ (DGAT1). **(B)** Impact of impaired LD biogenesis on chlamydial replication was evaluated by measuring bacterial yields expressed as inclusion forming unit per microliter (IFU/μl) in IFU assays, as detailed in Materials and Methods. Compared to *wt* cells, chlamydial replication was reduced in *dgat1/2* ^*-/-*^ whereas increased chlamydial yields were observed in *dgat1/2* ^*-/-*^ (DGAT1) compared to *dgat1/2* ^*-/-*^ (Control). Bars represent the mean and standard deviation. Data is representative of at least 3 independent experiments. Asterisks indicate statistically significant differences (***p<0.001; **p<0.01) as calculated by unpaired, two-tailed T-test. Input IFUs (mean ± standard deviation) were very similar for all cell types: *wt*, 19104 ± 1207; *dgat1/2* ^-/-^, 18758 ± 1008; *dgat1/2* ^*-/-*^ (Control), 18691 ± 1163; *dgat1/2* ^*-/-*^ (DGAT1) 18484 ± 1128.

Previous findings indicated that *C*. *trachomatis* replication is negatively impacted by pharmacological inhibition of neutral lipid synthesis with Triacsin C [28]. Based on those observations, our findings that LD expansion occurs during infection, and that lipid biosynthetic enzymes accumulate in LDs, we considered the possibility that the expansion of LDs was important for optimal generation of *C*. *trachomatis* infectious progeny. We addressed this by taking advantage of mouse embryo fibroblasts (MEF) derived from animals deficient for the diacylglycerol-acyltransferases 1 and 2 (DGAT1 and DGAT2) [79]. These cells are unable to synthesize triacylglycerols and thereby cannot generate LDs upon stimulation with oleic acid. We verified that even upon strong oleic acid stimulation (400 μM), *dgat1/2* double knock out (*dgat1/2* ^-/-^) MEFs were unable to generate LDs whereas wild type MEFs accumulated abundant LDs (Fig 8A). Complementation of DGAT1 expression in *dgat1/2* ^-/-^ double knock out cells restored the ability to produce LDs upon stimulation with oleic acid (Fig 8A). Similar attempts with stable DGAT2 expression in *dgat1/2* ^-/-^ double were unsuccessful, likely because overexpression of *DGAT2* is toxic to cells. We then verified that there were no significant differences in *C*. *trachomatis* entry in these cell types (data not shown) and proceeded to carry out IFU experiments to assess the number of infectious progeny generated. We found that generation of infectious progeny was impaired in *dgat1/2* ^-/-^ compared to wild type MEFs (Fig 8B) and reintroduction of DGAT1 in *dgat1/2* ^-/-^ restored LD expansion and increased the yields *C*. *trachomatis* infectious particles compared to control cells (Fig 8B). These findings strongly suggest that host triacylglycerols stored in LDs or their role in cellular lipid homeostasis is required for optimal chlamydial replication.

## Conclusions

This study provides a quantitative evaluation of the LD proteome in mammalian cells. Furthermore, we provide evidence for distinct changes to the LD proteome upon infection with the obligate intracellular pathogen *C*. *trachomatis*, suggesting that these organelles play a role in host-pathogen interactions. Consistent with this observation, genetic ablation of LDs led to reduced yields in infectious progeny generation within infected fibroblasts. While the mechanism underlying these observations are unknown, these findings support a role for LDs in multiple cellular processes including those triggered during infection ([33] and references therein). The finding that a subset of *C*. *trachomatis* Incs associate to LDs is particularly interesting, especially given that at least two of these Incs (Cap1 and CTL0882) strongly associate with LDs when expressed as transgenes in HeLa cells. We postulate that since these proteins are strategically located in the interphase between the parasitophorous vacuole and host cytoplasm/organelles, the may potentially play a role in manipulating LDs and host lipid homeostasis. In conclusion, our findings indicate that: *i) C*. *trachomatis* expresses proteins with affinity for both the inclusion membrane and LDs (this work and [28]) and *ii)* the LD proteome is modified during infection with *C*. *trachomatis*, including an increase in LD-associated host proteins involved in lipid metabolism and biosynthesis. Finally, the lack of LD formation in *dgat1/2* ^-/-^ cells correlates with impaired generation of infectious progeny, indicating that these neutral lipid-rich organelles contribute to optimal *C*. *trachomatis* growth.

## Supporting Information

S1 Fig Band profile and immunoblot analysis of HeLa LD fractions. **(A)** LD fractions from HeLa cells were separated by density gradient centrifugation and LD-associated proteins were acetone-precipitated after ether-extraction of the lipids to prepare LD protein extracts. Protein content was assessed by Bradford assays and equal amounts (5 μg/lane) of LD extracts and total HeLa lysates (T) were separated by 4–15% gradient SDS-PAGE. To visualize the band patterns, proteins were stained with Sypro-Orange. LD extracts showed a unique band pattern, compared to total lysates, as expected. Protein marker (M) and molecular weight values (kDa) are indicated [34]. **(B)** Proteins separated by SDS-PAGE were transferred onto nitrocellulose membranes and immunoblotted with the indicated antibodies. Primary antibodies PLIN3, PLIN2, ACSL3, ACSL4, NSDHL, PLPL2, AUP1 and FAF2 were used to confirm enrichment in LD-associated proteins. TRAP-α (endoplasmic reticulum, ER), Integrin-α2 (plasma membrane, PM) and GAPDH (cytosol) were used to assess contamination with other cell compartments.
(TIF)

S2 Fig Ectopic expression of fluorophore-tagged Cap1 and CTL0882 inclusion membrane proteins in the absence of oleic acid stimulation. Representative confocal images of HeLa cells transiently transfected with constructs encoding C-terminally tagged EGFP and N-terminally tagged Cherry fusions of Cap1 or CTL0882, as indicated, in the absence of stimulation with oleic acid. Cherry fusions displayed a punctate localization pattern whereas EGFP fusions showed a reticular localization pattern. Hoechst staining was used to visualize DNA (blue). Bar sizes are shown in the merge panels.
(TIF)

S3 Fig Ectopic expression of fluorescently labeled IncG in HeLa cells. Representative confocal images of HeLa cells transiently transfected with a construct encoding N-terminally tagged Cherry fusion of IncG, stimulated with oleic acid 200 μM for 8 h prior to fixation **(A)** or not **(B)**. In the presence of oleic acid stimulation, ectopically expressed Cherry-IncG was occasionally found co-localizing with LDs (stained with BODIPY 493/503, green channel), but much more frequently displaying a reticular pattern and no co-localization with LDs (see insets in **A** for a representative image of each localizations). In the absence of oleic acid stimulation, Cherry-IncG also showed a reticular localization pattern (see in **B**). Hoechst staining was used to visualize DNA (blue). Bar sizes are shown in the merge panels.
(TIF)

S1 Table All peptide identifications and label-free peak areas based on processing of the raw data in Rosetta Elucidator. The table includes peptide sequences, mass, charge, and retention time of the observed peptides, as well as the database search scores (PLGS and Mascot). Protein inference was performed based on the PeptideProphet algorithm, and the protein identification and descriptors are also reported. The peak areas are reported for each individual technical and biological replicate across all samples.
(XLSX)

S2 Table Quantitative data for proteins highly represented in LD extracts from uninfected and *C*. *trachomatis*-infected cells, 20 hpi. Listed are all proteins (n = 107) detected with > 3 peptides to match and highly reproducible quantitative data (within group % CV < 25% across 6 replicates per experimental condition) in LD extracts isolated from HeLa cells uninfected or infected with *C*. *trachomatis*, as determined by LC-MS/MS. Quantitative data for individual replicates as well as the mean and standard deviations (SD) for *C*. *trachomatis*-infected (20 hpi) and their corresponding uninfected control samples are provided (fmol μg^-1^). The number of peptides to match for each protein is indicated. Protein names, ID and description are according to Uniprot Protein knowledgebase.
(XLSX)

S3 Table Quantitative data for proteins highly represented in LD extracts from uninfected and *C*. *trachomatis*-infected cells, 40 hpi. Listed are all proteins (n = 107) detected with > 3 peptides to match and highly reproducible quantitative data (within group % CV < 25% across 6 replicates per experimental condition) in LD extracts isolated from HeLa cells uninfected or infected with *C*. *trachomatis*, as determined by LC-MS/MS. Quantitative data for individual replicates as well as the mean and standard deviations (SD) for *C*. *trachomatis*-infected (40 hpi) and their corresponding uninfected control samples are provided (fmol μg^-1^). The number of peptides to match for each protein is indicated. Protein names, ID and description are according to Uniprot Protein knowledgebase.
(XLSX)

S4 Table Proteins highly represented in LD extracts from uninfected and *C*. *trachomatis*-infected cells ranked by abundance. The expression levels for each protein listed in S2 and S3 Tables were averaged and ranked by decreasing abundance. Mean and standard deviation (SD) values are shown (fmol μg^-1^). The number of peptides to match for each protein is indicated. Protein names, ID and description are according to Uniprot Protein knowledgebase.
(XLSX)

S5 Table Select proteins detected in LD extracts grouped by gene ontologies. Listed are all proteins detected in LD extracts from uninfected and *C*. *trachomatis*–infected HeLa cells corresponding to select gene ontology (GO) categories analyzed in Fig 2 and Fig 4.
(XLSX)

S6 Table Fold changes of expression levels for proteins detected in LD extracts from *C*. *trachomatis-*infected cells with respect to uninfected controls. For each pair of samples (infected and their corresponding uninfected controls, 6 pairs for each 20 h and 40 h experiments), the ratio of expression levels in infected vs. uninfected was calculated in order to obtain the mean ratio and the average fold change for each time point. Reciprocal values were used for calculating the fold changes of down-regulated proteins. Proteins for which fold changes were > 1.5 or < -1.5 and with *p* values < 0.05 were considered as significantly changed and their gene ontologies retrieved from Uniprot.
(XLSX)

S7 Table List of chlamydial proteins enriched in LD extracts by LC-MS/MS. *C*. *trachomatis* proteins detected with >3 peptides to match and highly reproducible quantitative data (within group % CV < 25% across 6 replicates per timepoint) in LD extracts isolated from HeLa cells infected with *C*. *trachomatis*, as determined by LC-MS/MS, are listed (n = 3). Quantitative data for individual replicates as well as the mean and standard deviations (SD) are provided (fmol μg^-1^). The number of peptides to match for each protein is indicated. The primary locus encoding each protein is indicated according to the gene nomenclature for *C*. *trachomatis* L2 434/Bu reference strain (434/Bu column) and the corresponding ortholog for the widely used *C*. *trachomatis* serovar D UW-3/CX reference strain (UW-3/CX column). The abbreviated gene name, when available, is included. GI identifiers (assigned by NCBI), protein names and functional group are shown.
(XLSX)
