# Supplementary figures and images for: Chlamydia trachomatis Infection Leads to Defined Alterations to the Lipid Droplet Proteome in Epithelial Cells
Source: PLoS One. 2015 Apr 24;10(4):e0124630. doi: 10.1371/journal.pone.0124630 (PMC4409204; doi:10.1371/journal.pone.0124630)

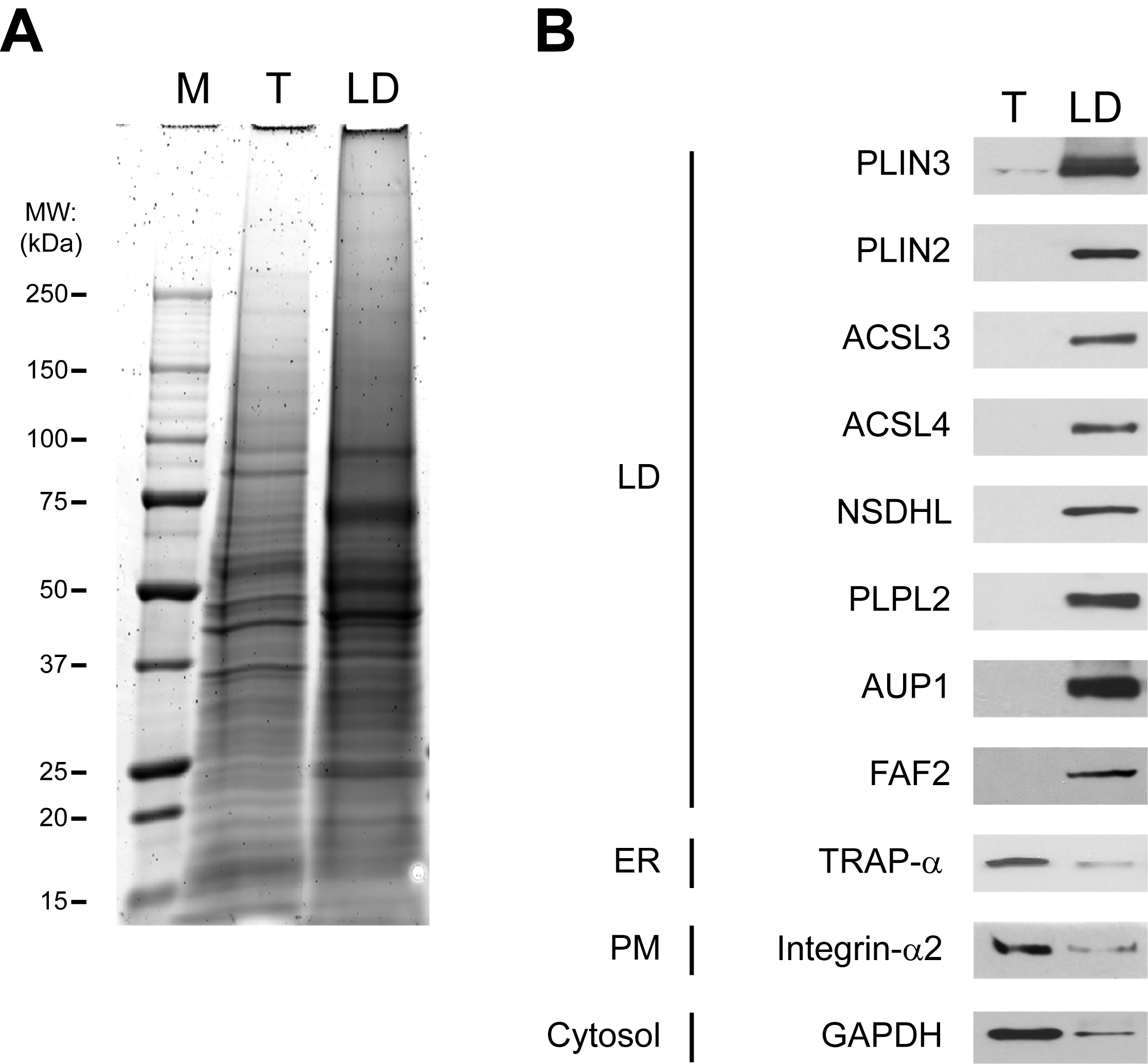

Supplement: S1 Fig — (A) LD fractions from HeLa cells were separated by density gradient centrifugation and LD-associated proteins were acetone-precipitated after ether-extraction of the lipids to prepare LD protein extracts. Protein content was assessed by Bradford assays and equal amounts (5 μg/lane) of LD extracts and total HeLa lysates (T) were separated by 4–15% gradient SDS-PAGE. To visualize the band patterns, proteins were stained with Sypro-Orange. LD extracts showed a unique band pattern, compared to total lysates, as expected. Protein marker (M) and molecular weight values (kDa) are indicated [34]. (B) Proteins separated by SDS-PAGE were transferred onto nitrocellulose membranes and immunoblotted with the indicated antibodies. Primary antibodies PLIN3, PLIN2, ACSL3, ACSL4, NSDHL, PLPL2, AUP1 and FAF2 were used to confirm enrichment in LD-associated proteins. TRAP-α (endoplasmic reticulum, ER), Integrin-α2 (plasma membrane, PM) and GAPDH (cytosol) were used to assess contamination with other cell compartments. (TIF) [file pone.0124630.s001.tif]

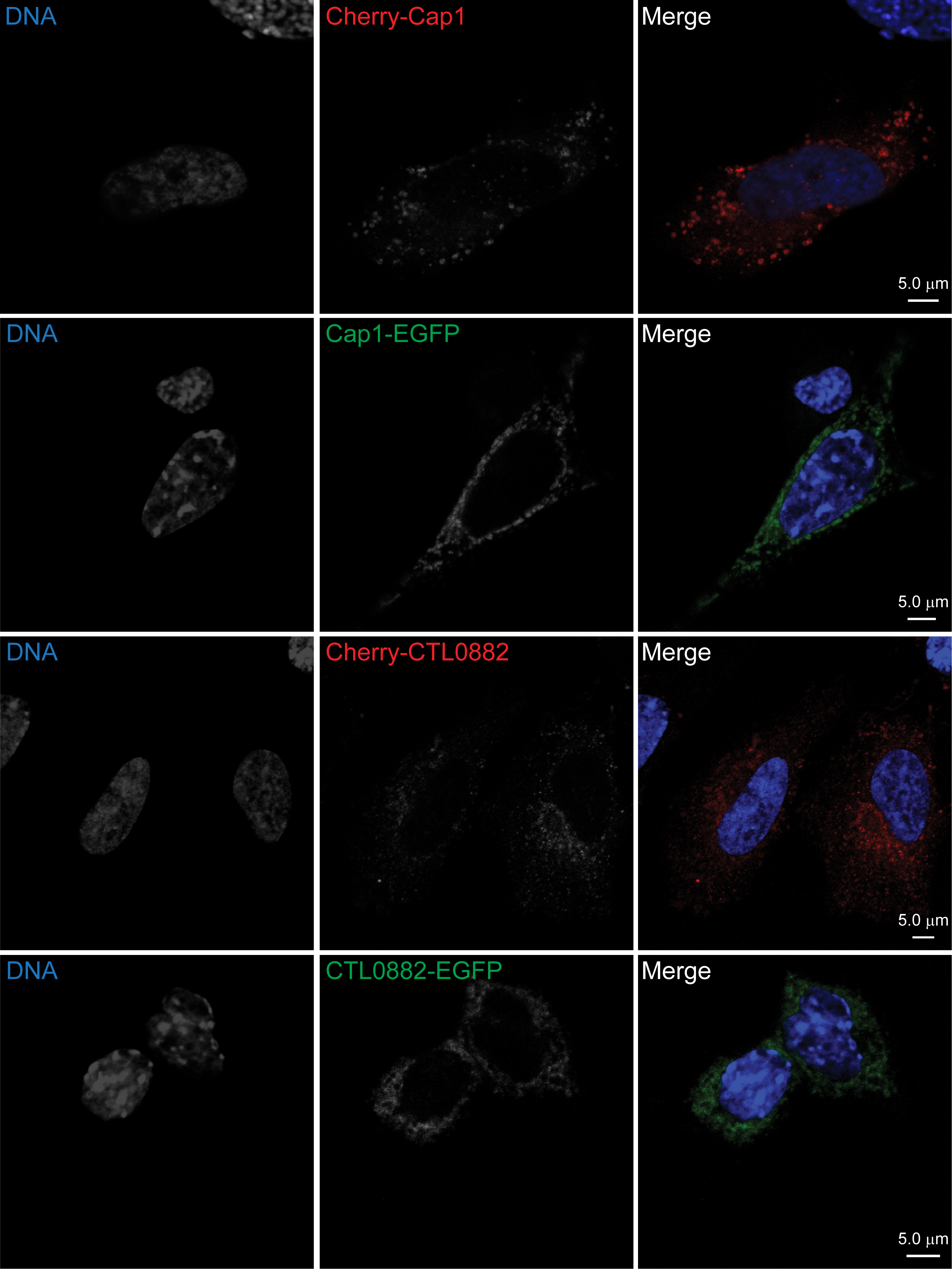

Supplement: S2 Fig — Representative confocal images of HeLa cells transiently transfected with constructs encoding C-terminally tagged EGFP and N-terminally tagged Cherry fusions of Cap1 or CTL0882, as indicated, in the absence of stimulation with oleic acid. Cherry fusions displayed a punctate localization pattern whereas EGFP fusions showed a reticular localization pattern. Hoechst staining was used to visualize DNA (blue). Bar sizes are shown in the merge panels. (TIF) [file pone.0124630.s002.tif]

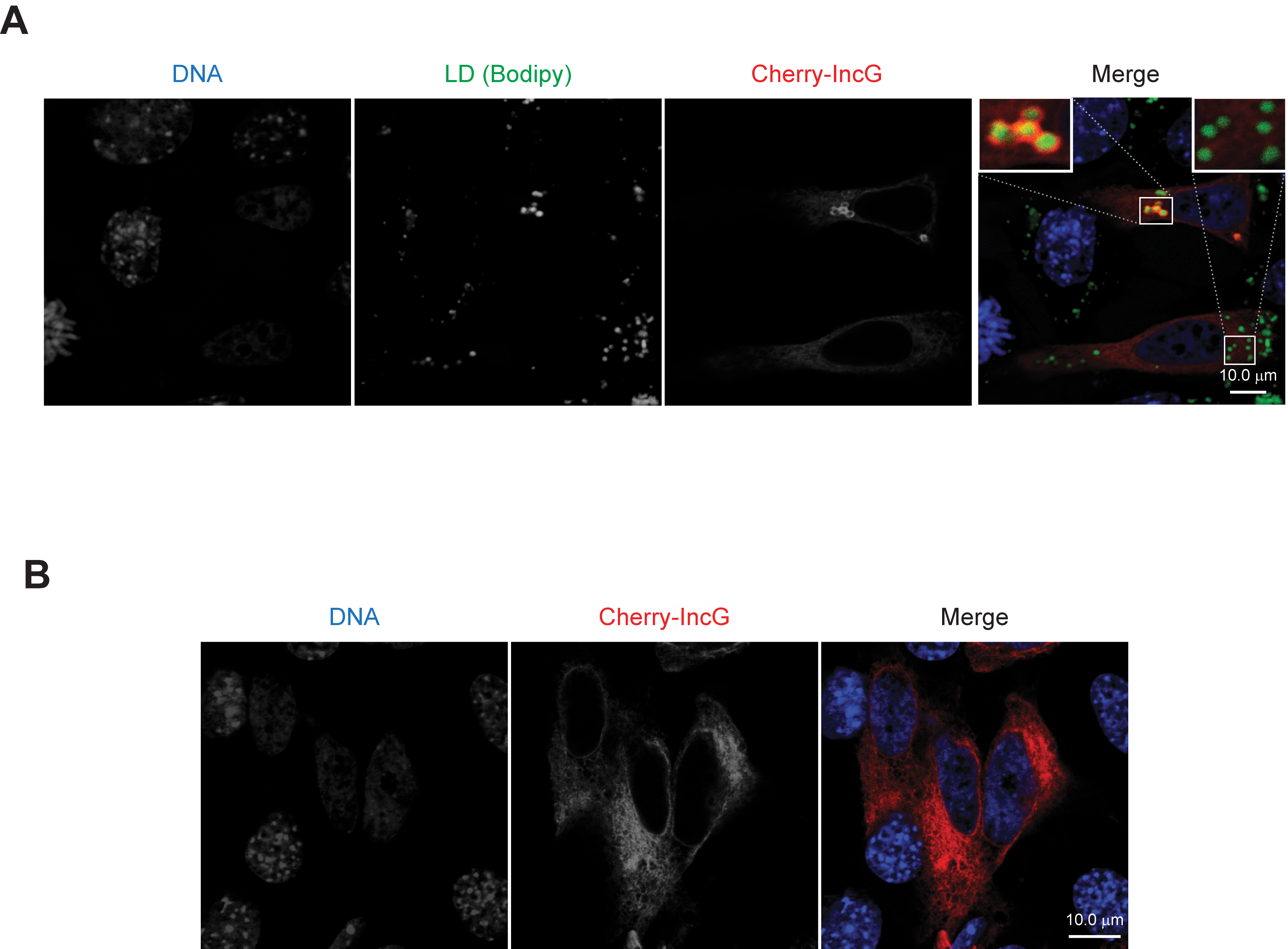

Supplement: S3 Fig — Representative confocal images of HeLa cells transiently transfected with a construct encoding N-terminally tagged Cherry fusion of IncG, stimulated with oleic acid 200 μM for 8 h prior to fixation (A) or not (B). In the presence of oleic acid stimulation, ectopically expressed Cherry-IncG was occasionally found co-localizing with LDs (stained with BODIPY 493/503, green channel), but much more frequently displaying a reticular pattern and no co-localization with LDs (see insets in A for a representative image of each localizations). In the absence of oleic acid stimulation, Cherry-IncG also showed a reticular localization pattern (see in B). Hoechst staining was used to visualize DNA (blue). Bar sizes are shown in the merge panels. (TIF) [file pone.0124630.s003.tif]
